# Robotic evaluation of articular laxity (REAL) classification: a new intraoperative knee soft-tissue laxity classification using ROSA robotic software

**DOI:** 10.1007/s00590-025-04265-w

**Published:** 2025-03-29

**Authors:** Eustathios Kenanidis, Nikolaos Milonakis, Alexandros Maslaris, Eleftherios Tsiridis

**Affiliations:** 1 Academic Orthopaedic Department, General Hospital Papageorgiou, Aristotle University of Thessaloniki School of Medicine, Thessaloniki, Greece; 2 Center for Interdisciplinary Research and Innovation (CIRI), Centre of Orthopaedic and Regenerative Medicine (CORE), Aristotle University of Thessaloniki, Thessaloniki, Greece

**Keywords:** ROSA, TKA, Robotic TKA, Total knee arthroplasty, Knee laxity, Knee balance, Functional alignment

## Abstract

**Purpose:**

Currently, there is no widely accepted method for measuring soft-tissue laxity and defining a balanced total knee arthroplasty (TKA). We aim to evaluate whether robotic technology can facilitate the categorization of intraoperative knee laxity.

**Methods:**

Our study was conducted in two phases. A senior surgeon performed imageless robotically assisted TKAs (ra-TKAs) using functional alignment. The first phase included 120 patients. Following the surgical approach, the medial and lateral soft-tissue laxity was recorded in extension and 90° flexion. The distribution of the difference and sum of laxities in extension and 90° flexion was assessed to classify laxity phenotypes. The second phase validated the classification in 102 additional ra-TKAs. Laxity phenotypes were evaluated at the start and end of the procedure.

**Results:**

Laxity difference followed a normal distribution, facilitating categorization into three groups, with a standard deviation of 2.5 mm. Three categories of mediolateral laxity severity difference were established: < 2.5 mm, 2.5-5 mm, and > 5 mm. These laxity groups were coded in extension as 1, 2, and 3 and in flexion as A, B, and C, respectively. Nine laxity phenotypes emerged from the combination of the extension and flexion categories (1A-C, 2A-C, and 3A-C). Phenotypes 1A and 1B were the most common at the operation’ beginning, while phenotypes 3B and 1C were the rarest. At the end of the operation, 93% were categorized as class 1A and 1B, defining the “balanced area”.

**Conclusion:**

Our study recognized nine intraoperative soft-tissue knee laxity phenotypes, potentially laying the groundwork for a surgical consensus on knee balancing.

**Supplementary Information:**

The online version contains supplementary material available at 10.1007/s00590-025-04265-w.

## Introduction

The variation in osteoarthritic knee phenotypes and their impact on selecting the most suitable total knee arthroplasty (TKA) method remains an ongoing research topic [1]. Different classification systems have been introduced to describe the coronal knee alignment [1, 2]. The coronal plane alignment knee classification (CPAK) is based on calculating the arithmetic hip-knee-ankle (HKA) angle and the joint line orientation [2]. This classification features nine phenotypes and is useful for evaluating pre-arthritic constitutional coronal alignment restoration but does not consider the deviation’s severity or ligament laxities [2]. Functional knee phenotypes (FKPs) provide a comprehensive patient anatomy evaluation [1]. This classification involves a femoral, a tibial, and a true HKA alignment angle, including the joint line convergence angle (JLCA). Beyond orientation, the FKP includes information about the deviation severity, with five subtypes per phenotype and 125 possible combinations [1]. While gender-specific differences in FKP phenotypes are primarily related to the femoral mechanical angle, the JLCA is gender-independent and mainly affected by bone morphology [3]. Recently, Hirschman et al. classified coronal knee alignment into neutral, normal, deviant, and aberrant types based on FKP, advancing towards a more personalized approach [4]. However, the authors acknowledged the need to comprehend knee phenotypic diversity further, including soft-tissue laxities [4]. It remains unclear whether knee soft-tissue laxity phenotypes exist during TKA and their influence on surgical decisions and outcomes. A recent robotically assisted TKA (ra-TKA) study indicated that soft-tissue laxity varies significantly among patients and cannot be predicted based on preoperative deformity [5]. Limited studies have correlated intraoperative knee laxity measurements with postoperative outcomes, showing inconsistent results [6–8].

We aimed to assess whether the knee laxity evaluation during ra-TKA could enhance intraoperative knee laxity categorization. In the first study phase, we aimed to measure and classify the soft-tissue knee laxity in full extension and at 90^0^ flexion during ROSA ra-TKA in 120 patients (Zimmer Biomet, Warsaw, IN, USA). All operations were performed by a senior surgeon using a gap-balancing technique. In the second study phase, we aimed to clinically validate the new classification in another 102 ra-TKAs performed by the same surgeon using the same surgical technique. Based on the classification, we aimed to determine the percentage of knees fully balanced at the end of the operation.

## Methods

This study was approved by the Academic Health Research Ethics Board, and all patients provided written informed consent before inclusion (122/20-02-2024).

### Phase 1: Measurement and classification of soft-tissue laxity

This phase involved 120 patients with end-stage knee osteoarthritis who underwent unilateral ra-TKA using the ROSA imageless option (Zimmer Biomet, Warsaw, IN). During the ROSA’s initial registration phase, we assessed the knees’ medial and lateral soft-tissue laxity and aimed to classify the laxities.

### Surgical technique

All procedures were performed by a senior surgeon, who employed a gap-balancing technique using functional alignment. Robotic procedures were conducted beyond the surgeon’s initial training curve. The same cemented posteriorly stabilized prosthesis (NexGen Legacy LPS Flex, Zimmer Biomet, Warsaw, IN) was used in all cases. Thigh tourniquet pressure was applied before the skin incision and maintained until skin closure. All patients received general anaesthesia.

Following a standard medial parapatellar approach, the menisci, cruciate ligaments, and osteophytes were removed, and the appropriate soft-tissue releases were performed. For mild varus or valgus knees, almost no releases were conducted. Releases to the mid-coronal tibial plane were done for severe varus knees, while severe valgus knees were treated according to Ranawat’s method [9]. The standard landmarks were identified according to the ROSA protocol. During the initial protocol assessment, the surgeon consistently applied varus and valgus stress forces with the knee in full extension and 90^o^ of flexion according to the manufacturer’s recommendations for all cases. The medial and lateral knee soft-tissue laxities were displayed in mm on the robotic touchscreen and recorded, indicating the maximum distance between femoral and tibial condyles under varus and valgus stress.

Afterwards, the surgeon performed the intraoperative planning to restore the native joint line obliquity and planned the proximal tibial cut to recreate the medial proximal tibial angle, with a boundary of 5° varus to 2° valgus. The tibial slope matched the native pre-arthritic medial tibial plateau slope. The distal femoral cut was around the medial side implant thickness (9 mm) to maintain the joint line height and parallel to the tibial resection to achieve a rectangular extension gap, restricted between 5° valgus and 3° varus. A HKA angle between 6° varus and 3° valgus was allowed. In cases lacking extension, supplementary 1–2 mm was added to the planned distal femoral cut. If recurvatum was present, first cuts were rather conservative. The planned bony resection depth was 19 mm, matching the implant thickness, with a target extension gap of 20–21 mm and residual laxities of 1–2 mm.

The necessary distal femur and proximal tibia resections were then performed, and the cuts’ accuracy was validated. A static spacer was used to evaluate the extension gap balance. If necessary, additional releases and recuts were performed until the extension gap was balanced. The lift-off manoeuvre assessed the femoral component’s size, height, and rotation. We aimed for a balanced flexion gap, with 1–2 mm residual laxity laterally and medially. The flexion gap was evaluated using a static spacer. Based on the plane, the femoral cuts were performed. Trial implants were inserted, and the overall lower limb alignment and tibial component rotation were assessed. Standard instruments were used to prepare the proximal tibial, and the permanent implants were fixed.

### Laxities measurements, evaluation and classification

The medial and lateral soft-tissue knee laxity measurements were taken during the initial ROSA protocol assessment. The difference and the sum of the medial and lateral soft-tissue laxities in full extension and at 90^0^ flexion were measured. We then investigated whether these values followed a normal distribution. If a normal laxity distribution was present, knees could be categorized into three groups based on the standard deviation (SD) from the mean. The first category would be defined by the limits between − 1SD and + 1SD around the mean, the second category by the limits between the + 1SD and + 2SDs (or − 1SD and − 2 SDs), and the third category by the limits above the second SD (or below the − 2SD) from the mean. Combining the laxity phenotype at full extension and 90^0^ flexion would determine the final classification.

### Phase 2: Validation of the classification

This phase involved 102 other ra-TKAs performed by the same surgeon using the same technique. In this group, the medial and lateral knee laxities were assessed in full extension and at 90^0^ of flexion at the beginning and end of the procedure after the final implants were placed. Based on the existing classification, the knees were classified at the beginning and end of the procedure.

### Statistical analysis

Standard methods were used for descriptive statistics. The Shapiro–Wilk and Kolmogorov–Smirnov tests were used to evaluate the data distribution normality. Statistical tests were two-tailed, and we set the alpha level at 0.05. Statistical analyses were performed using SPSS software (IBM, version 27.0).

## Results

### Phase 1

The mean patients’ age was 69.6 (± 8.69) years, with 89 females and 31 males. 51.7% of the knees were left, and 48.3% were right. There were 103 varus and 17 valgus knees. The mean sum (5.33 ± 2.38 mm) and difference (0.54 ± 2.72 mm) of the medial and lateral knee laxity in full extension followed a normal distribution [(Kolmogorov–Smirnov (KS) test 0.062, *p* = 0.2, Shapiro–Wilk (SW) test 0.987, *p* = 0.33 and KS test 0.06, *p* = 0.2, SW test 0.993, *p* = 0.805, respectively)]. The mean sum (6.57 ± 3.26 mm) of the medial and lateral knee laxity in 90° of flexion did not follow a normal distribution (KS test 0.079, *p* = 0.063, SW test 0.974, *p* = 0.021). The mean difference (0.203 ± 2.98 mm) between the medial and lateral laxities in 90^0^ of flexion followed a normal distribution (KS test 0.073, *p* = 0.175, SW test 0.98, *p* = 0.074). There was only one outlier for the extension and two for the flexion measurements.

As the distribution of the difference of the medial and lateral laxities followed normality in extension and flexion, we decided to use the difference of laxities to classify knee phenotypes. As previously described, we categorized the laxity into three groups based on the SD from the mean value. As the mean difference of knee laxity values in flexion and extension had an SD slightly over 2.5 mm, we used this value as the limit for categorization. The groups were labelled as 1, 2, and 3 for the extension values and A, B, and C for the 90^0^ flexion values accordingly. The first category (1 in extension/A in flexion) involved the knees with a difference of medial and lateral laxities below 2.5 mm. The second category (2 in extension/B in flexion) included the knees with a difference of laxities between 2.5 and 5 mm. The third category (3 in extension/C in flexion) involved knees with a difference of laxities greater than 5 mm. Combining the classification of a knee in both extension and flexion resulted in nine intraoperative laxity phenotypes of soft tissue and ligaments. This established the new REAL (Robotic Evaluation of Articular Laxity) classification (Table 1).

**Table 1:** The nine REAL (Robotic Evaluation of Articular Laxity) classification categories

REAL classification		Flexion categories		
		A (< 2.5 mm)	B (2.5–5 mm)	C (> 5 mm)
Extension categories	1 (< 2.5 mm)	1A	1B	1C
	2 (2.5–5 mm)	2A	2B	2C
	3 (> 5 mm)	3A	3B	3C

In this phase, most of the REAL phenotypes observed at the beginning of the operation belonged to the 1A class (45%). Other common initial intraoperative categories included classes 1B, 2A, and 2B, with the rarest being classes 3B and 1C. Table 2 presents the number and percentage of 120 knees categorized according to the REAL classification.

**Table 2:** A breakdown of the number and percentage of 120 knees in each category according to the REAL classification at the beginning of the operation

Class category	1A	1B	1C	2A	2B	2C	3A	3B	3C
Number (percentage)	54 (45)	19 (15.8)	3 (2.5)	20 (16.7)	13 (10.8)	3 (2.5)	2 (1.7)	4 (3.3)	2 (1.7)

### Phase 2

**Fig. 1:**
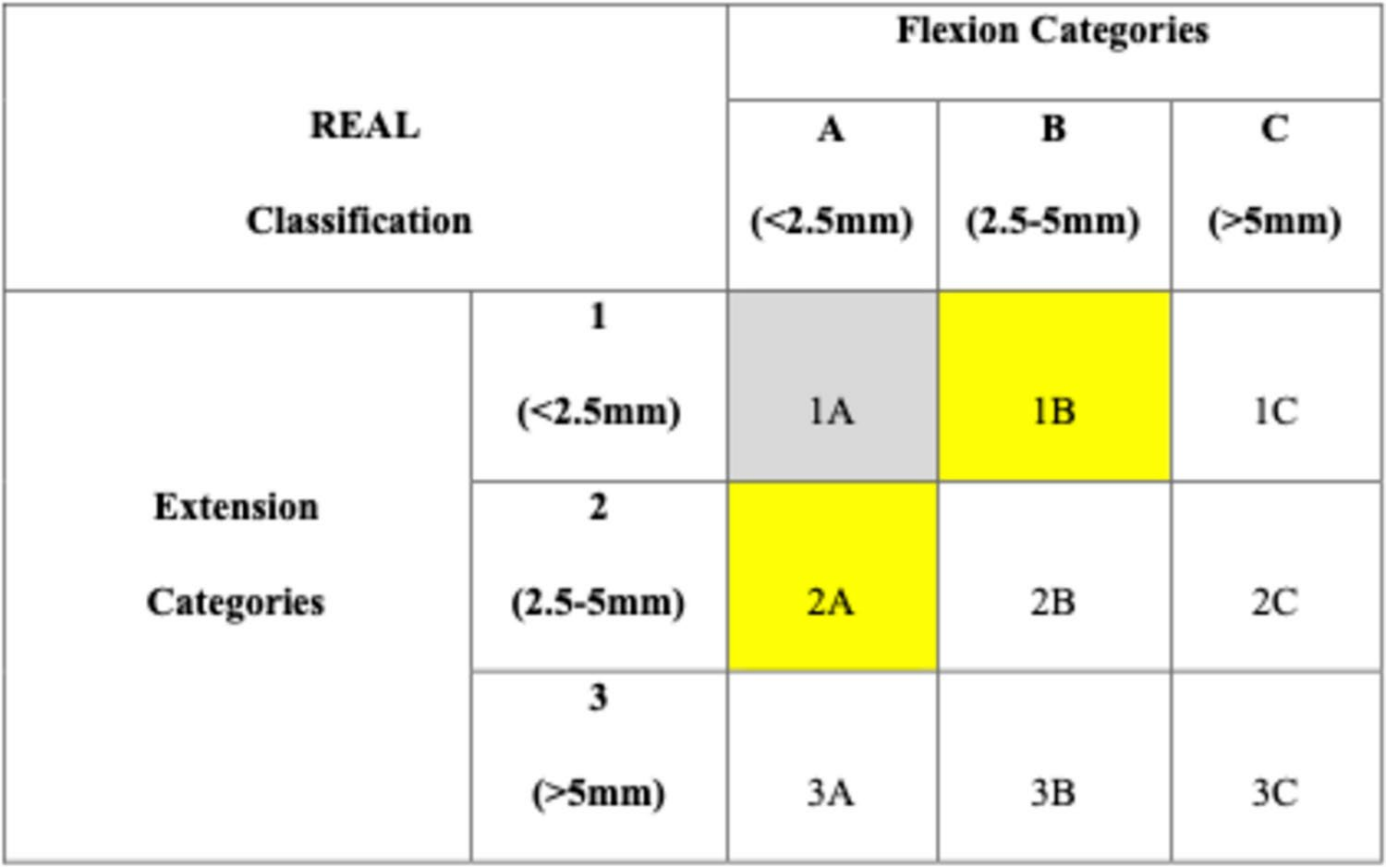
Visual representation of what is considered a “balanced knee” at the end of the procedure based on the evaluation with the REAL classification (grey colour balanced knee/yellow colour: almost balanced knee)

The mean patients’ age was 71.7 (± 9.68) years, with 73 females and 26 males. There were 38 left knees, 64 right knees, 89 varus knees, and 13 valgus knees. In this group, the most common REAL class during the initial intraoperative assessment was 1A, followed by 1B, 2A, and 2B. The Pearson correlation coefficient for the percentage of the REAL knee phenotypes at the initial intraoperative assessment between the study’s first and second phases was 0.039 (*p* = 0.7). At the end of the procedure, 68.6% of the knees were categorized as REAL class 1A, 22.5% as class 1B, and 2% as class 2A, resulting in a total of 93.1% for these categories. Table 3 shows the number and percentage of ra-TKAs classified in each REAL category at the beginning and the end of the operation for this group. The five cases categorized as 1C at the end of the procedure were initially categorized as 1A (2), 1B (1), 2B (1), and 2C (1). The two cases of 2A were initially also categorized as 2A, but the one case of 2B and one of 2C originated from 1A. Figure 1 presents a visual representation of what was considered a “balanced knee” at the end of the procedure.

**Table 3:** The number and percentage of ra-TKAs classified in each category at the beginning and end of the operation based on REAL classification for the group of 102 ra-TKAs in the study’s second phase

	REAL class	1A	1B	1C	2A	2B	2C	3A	3B	3C
Initial intraop assessment	Number (percentage**)**	43 (42.2)	24 (23.5)	2 (2)	12 (11.8)	10 (9.8)	8 (7.8)	–	1 (1)	2 (2)
Final intraop assessment		70 (68.6)	23 (22.5)	5 (4.9)	2 (2)	1 (1)	1 (1)	–	–	–

## Discussion

This study identified nine intraoperative knee laxity phenotypes based on the difference between the knee’s medial and lateral soft-tissue laxity in flexion and extension. For the first time, this functional classification considers the knee soft-tissue envelope rather than the bony structure. The REAL classification pertains to knees undergoing ROSA ra-TKA using functional alignment and a balancing technique. This classification may change, but it could serve as the basis for a widely accepted knee laxity classification.

Although achieving optimal knee balance during TKA is critical for a successful outcome [8], there is currently no consensus on what defines a balanced TKA [10]. Currently, surgical assessment of soft-tissue laxities is primarily subjective. CPAK and FKP provide a comprehensive assessment of patient anatomy concerning coronal knee alignment but do not offer insights into knee laxity [1, 2]. While intraoperative tools that measure ligament stresses are significant, a recent systematic review showed that Verasense pressure sensors and the manual balancing TKA technique resulted in similar clinical outcomes and reoperation rates. [11]. Recent advancements like ra-TKA could facilitate a more objective laxity assessment [5].

Significant variability exists in soft-tissue knee laxities [5, 12–17]. A recent ra-TKA study demonstrated that intraoperative soft-tissue knee laxities vary greatly among TKA patients, contributing 0–13 mm to the post-resection gap [14]. Additional research revealed considerable differences in medial soft-tissue contracture or laxity in varus knees [15–17]. Furthermore, the preoperative deformity does not reliably indicate the optimal bone resection and soft-tissue release required. [18]. The effects of bony resections on the components’ alignment and the resulting gap variability are still unclear [19, 20]. Identifying each patient’s unique bony characteristics and soft-tissue laxity at the procedure’s beginning may allow for a more personalized TKA approach.

Few studies have linked joint laxities at the procedure’s end with postoperative results, showing inconsistent results. Innocenti et al. showed that different laxity patterns in robotic-assisted medial unicompartmental knee arthroplasty with fixed-bearing yielded comparable results at the 2-year follow-up [6]. Conversely, having excessive lateral laxity in PS-TKA at an extension of over 4° degrees varus was linked to a lesser improvement in functional ability 1 year after surgery [7]. Besides, in a prospective study investigating 310 ra-TKAs, the intraoperatively measured joint gaps were associated with all functional outcomes two years after TKA [8].

The REAL classification may enhance communication among surgeons, support future research, and help define knee laxity. Combining bony and ligamentous knee classification may help surgeons understand knee phenotypes better, assess the necessary bone cuts, predict interventions to achieve final balance, and adopt an alignment philosophy per deformity. This study was based on ra-TKAs performed using restricted functional alignment, a technique ensuring a well-balanced knee in 98% of cases [21]. Future studies may evaluate which laxity classes to target for surgery using different alignment techniques.

This work has several limitations. It includes a limited yet reasonable number of patients, introducing the concept and emphasizing the need for further research. The classification was developed using intraoperative data from one experienced surgeon with a specific robotic system. While one surgeon ensures consistency in ligament tensioning, it may not reflect other surgeons’ techniques [22]. Interobserver reliability studies conducted by other surgeons with different alignment methods may support or challenge our findings. Currently, this classification relies on robotic technology, and its relevance to non-robotic cases requires further research. Furthermore, balancing goals can be deferred depending on the implant design used and surgeons’ technical preferences. Additionally, initial laxity evaluation occurred after the approach, anterior cruciate ligament and menisci release, potentially overestimating laxity by relieving knee strain. However, the ROSA robot allows assessment only at this stage. Intraoperative knee laxity evaluation can be affected by anaesthesia type, tourniquet use, and patient factors like obesity and previous hip surgeries. The intraoperative evaluation may overestimate coronal knee laxity; a preoperative assessment might better capture the patient’s natural knee tolerance [23].

Our study identifies nine intraoperative knee laxity types based on differences between the medial and lateral soft-tissue laxity during flexion and extension. The REAL classification applies to knees undergoing ROSA ra-TKA using functional alignment and balancing techniques. The REAL classification focuses on the knee soft-tissue envelope instead of bony structures to classify knees, potentially uniting surgeons on definitions and methods for knee balancing. Further evaluation is necessary, emphasizing the learning curve, validation studies for predicting postoperative outcomes, and its applicability in non-robotic cases to ascertain clinical relevance. These studies aim to standardize laxity measurements, justify classification thresholds, compare with existing systems, and address the implications of statistical assumptions.

## Supplementary Information

Below is the link to the electronic supplementary material.

Supplementary file1 (SAV 15 KB)

## Data Availability

Raw data supporting this study’s findings have been deposited together with other files during this submission (data sav.file).
